# Deciphering the constrained total energy expenditure model in humans by associating accelerometer-measured physical activity from wrist and hip

**DOI:** 10.1038/s41598-021-91750-x

**Published:** 2021-06-10

**Authors:** Rodrigo Fernández-Verdejo, Juan M. A. Alcantara, Jose E. Galgani, Francisco M. Acosta, Jairo H. Migueles, Francisco J. Amaro-Gahete, Idoia Labayen, Francisco B. Ortega, Jonatan R. Ruiz

**Affiliations:** 1 Carrera de Nutrición y Dietética, Departamento de Ciencias de la Salud, Facultad de Medicina, Pontificia Universidad Católica de Chile, Avenida Vicuña Mackenna, 4860 Macul, Santiago, Chile; 2 PROFITH “PROmoting FITness and Health Through Physical Activity” Research Group, Sport and Health University Research Institute (iMUDS), Department of Physical and Sports Education, Faculty of Sport Sciences, University of Granada, Crta Alfcar s/n, 18011 Granada, Spain; 3 Departamento de Nutrición, Diabetes y Metabolismo, Facultad de Medicina, Pontificia Universidad Católica de Chile, Santiago, Chile; 4 Department of Health, Medicine and Caring Sciences, Linköping University, 581 83 Linköping, Sweden; 5 Department of Physiology, Faculty of Medicine, University of Granada, Granada, Spain; 6 Institute for Innovation & Sustainable Development in Food Chain (IS-FOOD), IdisNA, Department of Health Sciences, Public University of Navarra, Campus de Arrosadía, Pamplona, Spain

**Keywords:** Endocrine system and metabolic diseases, Obesity, Homeostasis, Weight management

## Abstract

The constrained total energy expenditure (TEE) model posits that progressive increases in physical activity (PA) lead to increases in TEE; but after certain PA threshold, TEE plateaus. Then, a compensatory reduction in the expenditure of non-essential activities constrains the TEE. We hypothesized that high PA levels as locomotion associate with a compensatory attenuation in arm movements. We included 209 adults (64% females, mean [SD] age 32.1 [15.0] years) and 105 children (40% females, age 10.0 [1.1] years). Subjects wore, simultaneously, one accelerometer in the non-dominant wrist and another in the hip for ≥ 4 days. We analyzed the association between wrist-measured (arm movements plus locomotion) and hip-measured PA (locomotion). We also analyzed how the capacity to dissociate arm movements from locomotion influences total PA. In adults, the association between wrist-measured and hip-measured PA was better described by a quadratic than a linear model (Quadratic-R^2^ = 0.54 vs. Linear-R^2^ = 0.52; *P* = 0.003). Above the 80th percentile of hip-measured PA, wrist-measured PA plateaued. In children, there was no evidence that a quadratic model fitted the association between wrist-measured and hip-measured PA better than a linear model (R^2^ = 0.58 in both models, *P* = 0.25). In adults and children, those with the highest capacity to dissociate arm movements from locomotion—i.e. higher arm movements for a given locomotion—reached the highest total PA. We conclude that, in adults, elevated locomotion associates with a compensatory reduction in arm movements (probably non-essential fidgeting) that partially explains the constrained TEE model. Subjects with the lowest arm compensation reach the highest total PA.

## Introduction

The constrained total energy expenditure (TEE) model posits that, in humans and other endothermic species, TEE is homeostatically constrained within a narrow range^1^. The model predicts that, within low-to-moderate physical activity (PA) levels, increases in PA lead to increases in TEE. Yet after reaching higher PA levels, further increases in PA do not increase TEE^1^. Instead, high PA levels would entail reallocation of the constrained TEE budget from non-essential physiological activities (e.g. excessive innate immunity) to PA. This energy trade-off may explain some health benefits of regular PA^2^. For example, regular PA would decrease the energy available for inflammation, thus preventing diseases associated with chronic systemic inflammation^3^.

In adults, the model was initially supported by data showing similar TEE among populations with markedly different PA levels^4,5^. But more compelling evidence was obtained from a cross-sectional analysis of 332 adults with a wide range of PA patterns^6^. Therein, PA was measured by hip-worn accelerometers, and TEE by doubly labeled water. Among subjects with PA levels below the ~ 70th percentile of their measured PA span, PA was directly associated to TEE. Nevertheless, among subjects with PA levels above such threshold, PA and TEE were unrelated; higher PA levels were thus not associated with higher TEE^6^. This observation supports that TEE was constrained, and suggests that the TEE budget should have been reallocated to sustain such a high PA level. The authors hypothesized that the energy expended in non-muscular activities (e.g. immunity, reproduction) was partially reallocated to PA^6^.

Data comparing children from industrialized populations (US/UK; n = 44) and a forager-horticulturalist population (Shuar; n = 44) suggest that the model also operates during childhood^7^. In that study, PA level was measured by hip-worn accelerometers, and TEE by doubly labeled water. Although the Shuar children had higher PA levels than the US/UK children, TEE was not different between groups. Immune activity was also higher among Shuar children. Thus, to sustain PA levels and immune activity in Shuar children, a TEE reallocation comprising reductions in the energy expended in other physiological activities should have occurred.

Taken together, studies using hip-worn accelerometers to measure PA support the constrained TEE model in adults and children. But how this TEE constraint is achieved in face of high hip-measured PA levels is unknown. The reallocation of energy from non-essential, non-muscular physiological activities has been proposed^2,6,7^. It may also involve a compensatory decrease in muscular activities that hip-worn accelerometers cannot detect, such as arm movements^8^. Perhaps, above certain hip-measured PA (mostly determined by locomotion), a compensatory decrease in non-essential arm movements (e.g. fidgeting) helps to maintain TEE within its limits. Evidence in adults suggests that when hip-measured PA is zero (no locomotion), there are still ~ 600 kcal/d theoretically attributed to PA. This energy expenditure may partially correspond to non-essential arm movements that can be avoided in case of elevated locomotion. This compensation, along with the reallocation of energy expended in other non-essential activities, may have evolved to allow humans to maintain the long walking distances required for survival in the past^5^.

To test such hypothesis, we analyzed cross-sectional data from adults and children that worn—simultaneously—accelerometers in the hip and the non-dominant wrist. We hypothesized that, at low hip-measured PA levels, wrist-measured PA and hip-measured PA are directly associated; nevertheless, beyond a certain hip-measured PA threshold, the association plateaus. This would suggest that high levels of locomotion lead to reductions in arm movements, thus partially explaining the constrained TEE model. The aim of the study was to analyze the association between wrist-measured PA and hip-measured PA in adults and children.

## Results

### Association patterns between wrist-measured PA and hip-measured PA

We conducted a secondary analysis of the data of 209 adults from two clinical trials (NCT02365129^9^ and NCT03334357)^10^, and of 105 children from another clinical trial (NCT02295072)^11^ (see Methods, "Study design and subjects" subsection, for details). All subjects wore—simultaneously—one accelerometer in the non-dominant wrist and another in the right hip for ≥ 4 days. Table 1 shows the participant's main characteristics. The raw data from the accelerometers were processed to remove the gravity and noise components; Euclidean norm minus one *G* were then calculated in 5-s epochs, and PA was expressed in milli-gravitational units (m*g*; see Methods, "Physical activity" subsection, for details)^12,13^. To determine the shape of the association between wrist-measured PA and hip-measured PA, we first calculated the R^2^ for linear and quadratic regression models. Then, we iteratively removed subjects at low deciles of hip-measured PA to assess the association with wrist-measured PA above increasing hip-measured PA thresholds.

**Table 1:** Characteristics of the subjects.

	Adults	Children
n	209	105
Males/Females, n	75/134	63/42
Age, years	32.1 [15.0] (18.2–66.0)	10.0 [1.1] (7.9–11.9)
Weight, kg	72.4 [16.2] (45.0–123.7)	56.2 [11.0] (29.9–84.7)
Height, m	1.67 [0.08] (1.48–1.95)	1.44 [0.08] (1.23–1.66]
Body mass index, kg/m^2^	25.5 [4.4] (17.2–39.4)	26.9 [3.7] (19.7–37.8)
Fat mass, kg	26.8 [8.9] (10.0–51.7)	24.6 [6.7] (12.1–43.6)^B^
Lean mass, kg	42.2 [10.5] (22.7–73.7)	29.2 [5.2] (16.3–46.1)^B^
Resting metabolic rate, kcal/day	1466 [337] (723–2672)^A^	Not measured
**Hip accelerometer**		
Physical activity, m*﻿g*	11.5 [3.2] (3.0–21.8)	16.6 [3.9] (7.5–26.7)
Valid data, days	6.7 [0.5] (4.0–8.0)	6.9 [0.4] (4.0–8.0)
Non-wear time, h/day	0.34 [0.42] (0.00–1.98)	0.19 [0.20] (0.00–0.85)
**Wrist accelerometer**		
Physical activity, m*g*	24.1 [5.7] (10.2–42.4)	39.5 [8.7] (21.7–65.0)
Valid data, days	6.7 [0.5] (4.0–8.0)	6.9 [0.4] (5.0–8.0)
Non-wear time, h/day	0.30 [0.41] (0.00–2.47)	0.17 [0.20] (0.00–1.07)

Data are mean [standard deviation] (minimum − maximum).

^A^n = 169; ^B^n = 104.

**Figure 1:**
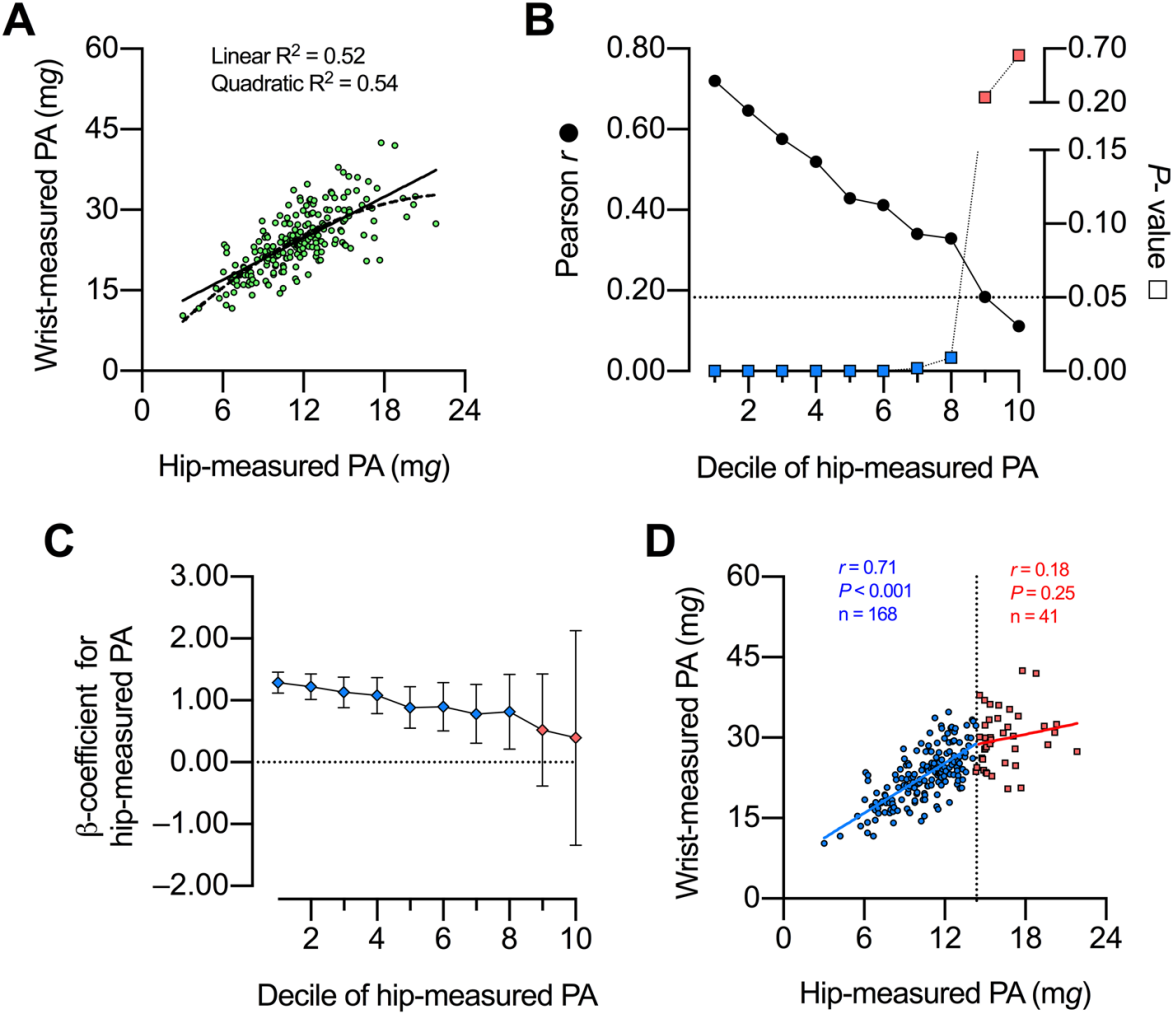
Association between wrist-measured and hip-measured physical activity [PA] in adults. (**A**) Quadratic [dashed line] and linear [solid line] regression models. (**B**) Pearson *r* [circles] and *P* values [squares; blue for statistically significant, and red for non-significant] for the associations between wrist-measured PA and hip-measured PA that include subjects from different deciles of hip-measured PA until the 10th decile; e.g. values for decile 9th indicate a correlation that includes subjects from the 9th to the 10th deciles. (**C**) Unstandardized ß-coefficients and 95% confidence intervals for the linear regression between wrist-measured PA vs. hip-measured PA that include subjects spanning different deciles of hip-measured PA, as explained in B. Blue symbols indicate significantly different from zero; red symbols indicate non-significantly different from zero (**D**) Threshold of hip-measured PA [dotted line; 14.33 mg] beyond which the association with wrist-measured PA becomes non-significant. n = 209.

For adults, the association between wrist-measured PA and hip-measured PA showed a higher R^2^ using a quadratic model than a linear model (Fig. 1A). The extra sum-of-squares F test confirmed that the quadratic model fitted better the data than the linear model (F [DFn, DFd] = 9.104 [1, 206]; *P* = 0.003, n = 209). When all adults were considered, wrist-measured PA and hip-measured PA correlated directly (Pearson *r* = 0.72, *P* < 0.001, n = 209). Nevertheless, the correlation became progressively weaker by iteratively excluding the lowest hip-measured PA deciles, becoming non-significant after the deciles 1st-8th had been excluded (Fig. 1B). Similarly, the unstandardized ß-coefficient [95% CI] of the linear regression between wrist-measured PA and hip-measured PA was not different from zero after excluding the deciles 1st-8th (0.52 [− 0.38–1.43], n = 41; Fig. 1C). We thus identified the 80th percentile of hip-measured PA (i.e. 14.33 m*g*) as a threshold beyond which there is no evidence of an association between wrist-measured PA and hip-measured PA (Fig. 1D). The association between wrist-measured PA and hip-measured PA below such a threshold was statistically significant, with a *y*-intercept [95% CI] different from zero (6.59 m*g* [4.05–9.13]; Fig. 1D). We then tested whether the lack of evidence for an association observed beyond the 80th percentile threshold was explained by the small sample size (n = 41). To that end, we analyzed the association between wrist-measured PA and hip-measured PA including the 41 adults with the lowest hip-measured PA. The association was significant (Pearson *r* = 0.51, *P* = 0.001, n = 41), suggesting that the sample size did not explain the effect observed beyond the 80th percentile threshold.

**Figure 2:**
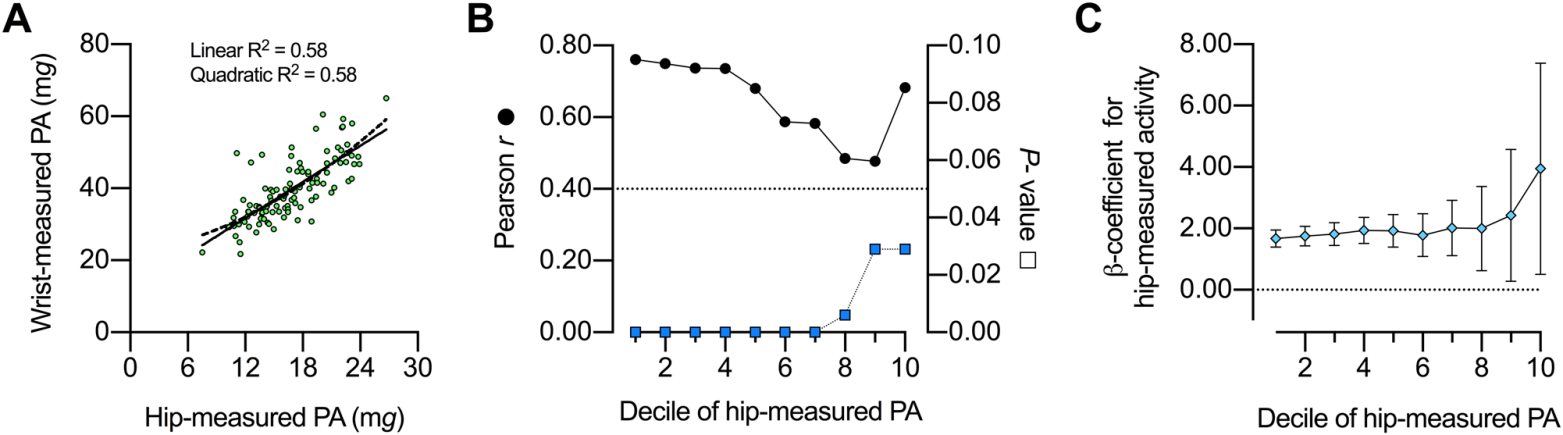
Association between wrist-measured and hip-measured physical activity [PA] in children. (**A**) Quadratic [dashed line] and linear [solid line] regression models. (**B**) Pearson *r* [circles] and *P* values [squares] for the associations between wrist-measured PA and hip-measured PA that include subjects from different deciles of hip-measured PA until the 10th decile; e.g. values for decile 9th indicate a correlation that includes subjects from the 9th to the 10th deciles. (**C**) Unstandardized ß-coefficients and 95% confidence intervals for the linear regression between wrist-measured PA vs. hip-measured PA that include subjects spanning different deciles of hip-measured PA, as explained in B. n = 105.

For children, the association between wrist-measured PA and hip-measured PA showed a similar R^2^ using a quadratic model or a linear model (Fig. 2A). The extra sum-of-squares F test confirmed that there was no evidence that the quadratic model fitted better the data than the linear model (F [DFn, DFd] = 1.349 [1, 102]; *P* = 0.248, n = 105); since the linear model was simpler, this was deemed the preferred model. When all children were considered, wrist-measured PA and hip-measured PA correlated directly (Pearson *r* = 0.76, *P* < 0.001, n = 105). Notably, the correlation remained significant when the lowest hip-measured PA deciles were iteratively excluded; indeed, when 1st-9th deciles had been excluded, wrist-measured PA and hip-measured PA were still associated (Pearson *r* = 0.68, *P* = 0.029, n = 10; Fig. 2B). Similarly, the unstandardized ß-coefficient of the linear regression between wrist-measured PA and hip-measured PA was always different from zero (Fig. 2C). Therefore, we did not observe any hip-measured PA threshold beyond which wrist-measured PA plateaued. The association between wrist-measured PA and hip-measured PA had a *y*-intercept [95% CI] different from zero (11.59 m*g* [6.81–16.37], n = 105; Fig. 2A).

### Dissociation of locomotion and arm movements at high total PA

We conducted complementary analyses to study how the capacity to dissociate arm movements from locomotion influences total PA. When accelerometers are worn on the hip, they mostly detect accelerations from activities that include locomotion (e.g. walking), but cannot detect accelerations from arm movements. In contrast, when accelerometers are worn on the wrist, they detect accelerations from activities that include locomotion as well as arm movements (e.g. arms swing during locomotion, occupational activities, fidgeting). As an approach to isolate the accelerations produced by arm movements, we adjusted wrist-measured PA for hip-measured PA (see Methods, "Statistical analyses" subsection, for details). This new index—wrist-measured PA_ADJ_—is thus a surrogate of the accelerations produced by arm movements. Total PA was then calculated by adding hip-measured PA (i.e. locomotion) to wrist-measured PA_ADJ_ (i.e. arm movements).

**Figure 3:**
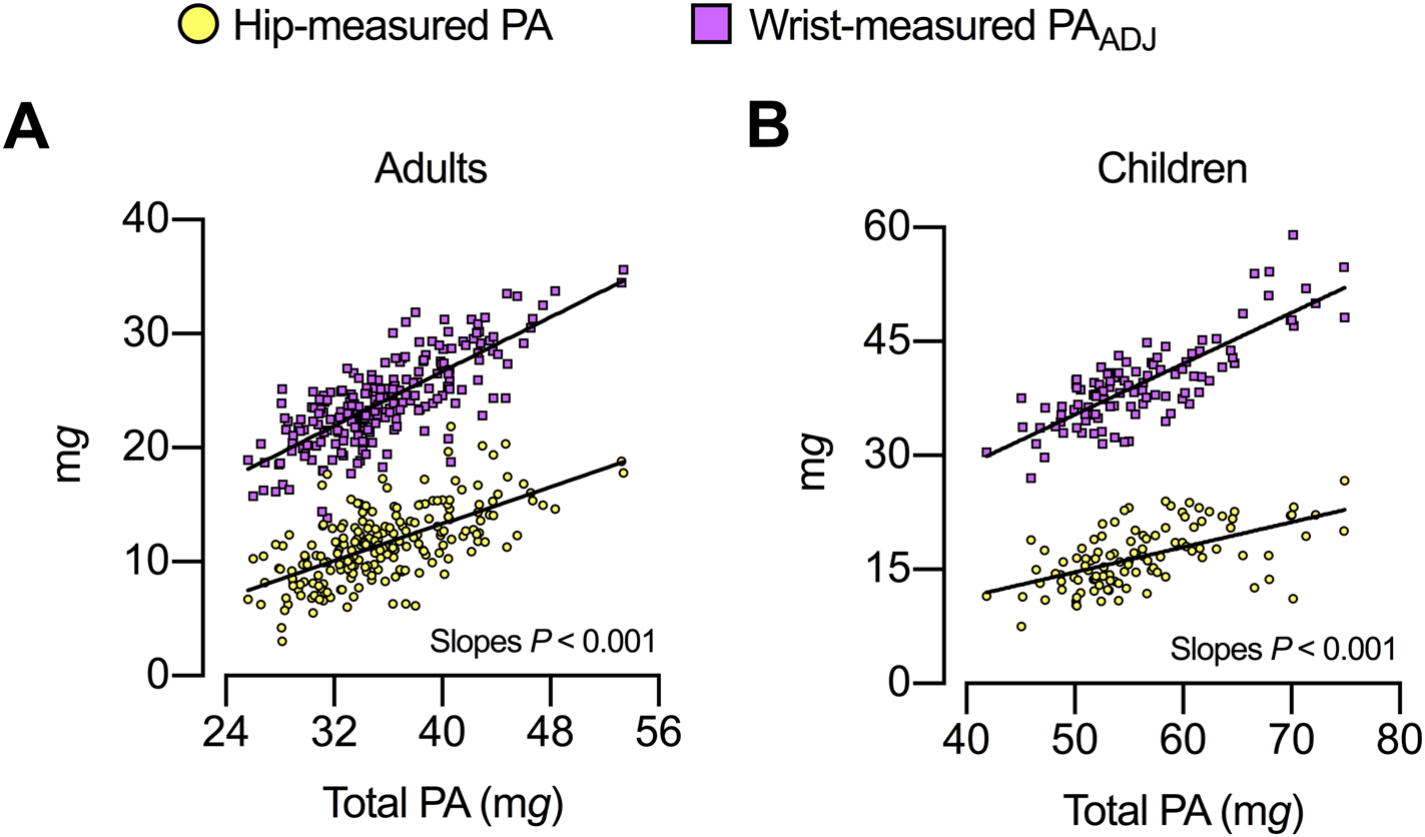
Association between total physical activity [PA] and its components in (**A**) adults and (**B**) children. Wrist-measured PA_ADJ_ represents wrist-measured PA adjusted for hip-measured PA. Adults n = 209; children n = 105.

In adults, as expected, hip-measured PA and wrist-measured PA_ADJ_ associated directly to total PA (Pearson *r* = 0.63 and 0.77, respectively; *P* < 0.001, n = 209). Notably, the slope [95% CI] of the linear regression for wrist-measured PA_ADJ_ (vs. total PA) was steeper than the slope of the linear regression for hip-measured PA (0.59 [0.52–0.66] vs. 0.40 [0.33–0.47], respectively; F [DFn, DFd] = 15.74 [1, 414]; *P* < 0.001, n = 209; Fig. 3A). In children, also as expected, hip-measured PA and wrist-measured PA_ADJ_ associated directly to total PA (Pearson *r* = 0.57 and 0.81, respectively; *P* < 0.001, n = 105). The slope [95% CI] of the linear regression for wrist-measured PA_ADJ_ (vs. total PA) was also steeper than the slope of the linear regression for hip-measured PA (0.67 [0.57–0.76] vs. 0.32 [0.23–0.42], respectively; F [DFn, DFd] = 27.26 [1, 206]; *P* < 0.001, n = 105; Fig. 3B).

### Resting metabolic rate is unrelated to PA

**Figure 4:**
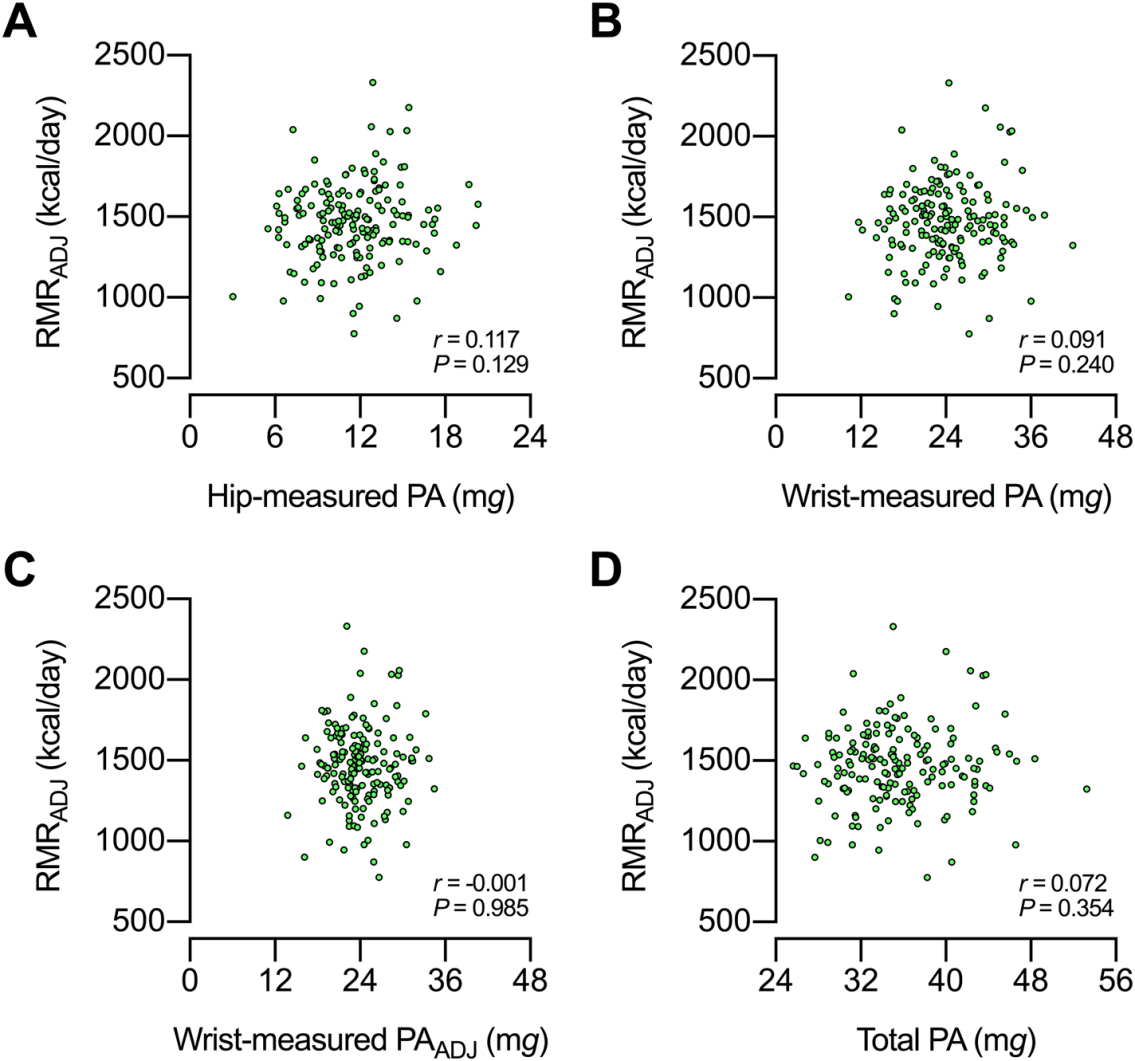
Association between resting metabolic rate [RMR] and physical activity [PA] in adults. RMR adjusted for lean mass [RMR_ADJ_] was associated with (**A**) hip-measured PA, (**B**) wrist-measured PA, (**C**) wrist-measured PA adjusted for hip-measured PA [wrist-measured PA_ADJ_], or (**D**) total PA [hip-measured PA plus wrist-measured PA_ADJ_]. n = 169.

We analyzed the association between resting metabolic rate (RMR) and the indexes of PA in adults. For analyses, RMR was first adjusted for lean mass (RMR_ADJ_). There was no evidence for an association of RMR_ADJ_ vs. hip-measured PA, wrist-measured PA, wrist-measured PA_ADJ_, or total PA (Fig. 4).

### Effects of sex and trial

Our databases included males and females. To ascertain how sex influenced our results, we repeated our main analyses stratified by sex. Table S1 shows the main characteristics of the adults and children stratified by sex. In adults, wrist-measured PA and hip-measured PA were similar in males and females (Table S1). In male adults, a quadratic model fitted better the association between wrist-measured PA and hip-measured PA than a linear model (F [DFn, DFd] = 13.69 [1, 72]; *P* < 0.001, n = 75; Fig. S1A). Nevertheless, in female adults, there was no evidence for such an effect (F [DFn, DFd] = 0.002 [1, 131]; *P* = 0.966, n = 134; Fig. S1B). These data indicate that our main findings in adults are mostly explained by males. In children, males had higher wrist-measured PA and hip-measured PA than females (Table S1). In male children, there was no evidence that a quadratic model fitted better the data than a linear model (F [DFn, DFd] = 1.602 [1, 60]; *P* = 0.210, n = 63; Fig. S1C). This was also the case for female children (F [DFn, DFd] = 1.162 [1, 39]; *P* = 0.287, n = 42; Fig. S1D). Our main results in children thus appear independent of sex.

Finally, our adult sample included a clinical trial in young adults (18–25 years; NCT02365129)^9^ and another in middle-age adults (45–65 years; NCT03334357)^10^. To ascertain how the characteristics of these trials influenced our results, we repeated our main analyses stratified by trial (young adults, middle-age adults). Table S2 shows the main characteristics of the adults stratified by trial. Middle-age adults had higher wrist-measured PA and hip-measured PA than young adults (Table S2). In young adults, a quadratic model fitted better the association between wrist-measured PA and hip-measured PA than a linear model (F [DFn, DFd] = 7.780 [1, 140]; *P* = 0.006, n = 143; Fig. S2A). In middle-age adults, a quadratic model also trended to fit better the association between wrist-measured PA and hip-measured PA than a linear model (F [DFn, DFd] = 2.709 [1, 63]; *P* = 0.104, n = 66; Fig. S2B). These findings suggest the trial had no major impact in our main results.

## Discussion

In humans, TEE is proposed to be maintained within a narrow physiological range, thus constraining excessive elevations induced by PA^5–7^. This constraint may have evolved as a mechanism to allow humans and other species to increase foraging effort without requiring greater dietary intake^1^. Thus, the energy to sustain foraging or other PA would be obtained by reallocating expended energy from other non-essential physiological activities^1,2^. What those “other” activities are is unknown. Herein, we tested the hypothesis that arm movements may be involved. We found that, in adults, the higher the PA measured by hip-worn accelerometers the higher the PA measured by wrist-worn accelerometers; however, beyond a certain hip-measured PA level, wrist-measured PA plateaued. This suggests that at high levels of locomotion (i.e. hip-measured PA), a compensation that reduces arm movements ensues. We speculate that this compensation limits the energy expended by non-essential PA (e.g. fidgeting) to reallocate such energy to locomotion, and may partly explain the resistance to weight loss of PA interventions^14^. In children, however, the direct association between wrist-measured PA and hip-measured PA did not plateau. Thus, our data do not support a compensation involving arm movements during childhood.

By simultaneously wearing accelerometers in the hip and the wrist, we intended to discriminate different components of the total PA. In adults, we observed that wrist-measured PA and hip-measured PA were directly associated. A quadratic model fitted the data better than a linear model. This indicates that as hip-measured PA increases, the increases in wrist-measured PA are progressively attenuated. Previous studies have reported linear associations between wrist-measured PA and hip-measured PA^15,16^. Nevertheless, whether a quadratic regression fitted better the data than a linear regression does not seem to have been tested. We thus replicated and then re-analyzed the scatterplots by Kamada et al. (Fig. 1A in ref.^15^) and Dieu et al. (Fig. 2 in ref.^16^). In the data by Kamada et al., which included 94 elderly women^15^, a quadratic model trended to fit better the data than a linear model (R^2^ = 0.60 and 0.58, respectively; F [DFn, DFd] = 3.752 [1, 91]; *P* = 0.055). This agrees with our current findings. In the data by Dieu et al., which included 40 young adults^16^, there was no evidence that a quadratic model fitted the data better than a linear model (R^2^ = 0.78 in both models; F [DFn, DFd] = 0.849 [1, 37]; *P* = 0.362).

At least two factors may explain the discrepancy of the findings from the data by Dieu et al.^16^ vs. our findings and those from the data by Kamada et al.^15^. First, the sample size by Dieu et al.^16^ was the smallest. Second, PA data by Dieu et al. was obtained during 1 day of measurement^16^, whereas we obtained data from at least 4 days (minimum 4 days, maximum 8 days); Kamada et al. measured subjects for 7 days^15^. Therefore, it is possible that the compensation in arm movement is not evident within a single day, but only over longer periods (≥ 4 days).

In adults, Pontzer et al.^6^ identified a hip-measured PA threshold beyond which TEE plateaus. Herein we used a similar analytical approach to identify a hip-measured PA threshold beyond which wrist-measured PA plateaus. We found this threshold at the 80th percentile of the hip-measured PA of our sample distribution (14.33 m*g*). We speculate that beyond this threshold a compensation that reduces non-essential arm movement—e.g. fidgeting—ensues to constrain TEE (note that arms swinging for locomotion would continue increasing). This supports the previous hypothesis for a compensatory reduction in non-exercise activity thermogenesis^17^. In contrast, we found that, below the threshold, wrist-measured PA and hip-measured PA were directly associated. Of note, the *y*-intercept of the association was different from zero. This indicates that when hip-measured PA is zero (no locomotion), a remaining wrist-measured PA is expected (e.g. fidgeting, occupational activities). In agreement, Pontzer et al.^6^ observed that when hip-measured PA was zero, the energy expenditure theoretically attributed to PA was ~ 600 kcal/day. They thus proposed that other non-muscular physiological activities were responsible for those 600 kcal/day. Our current data suggest that arm movement may be at least partially responsible. Arm movements—along with the thermic effect of food—may also explain why the ratio TEE/RMR (so named *PAL: Physical Activity Level*) reaches 1.2 in non-ambulant subjects^18^. But the proportion of those 600 kcal/day explained by arm movements cannot be ascertained with our data. Previous evidence demonstrates that fidgeting increases TEE by 0.6 kcal/min (46%) while sitting^19^. Also, analyses using calorimetric chambers have shown that spontaneous PA—with fidgeting as a major component—accounts for 100–800 kcal/day^20^. The energy expended in fidgeting, which is a non-essential PA, might thus represent an important proportion of the energy available for reallocation.

Previous evidence obtained during a 20-week running competition also suggests an energy reallocation from arm movements to locomotion^21^. In that study, TEE was measured by doubly labeled water, and then parsed into its components: RMR (measured by indirect calorimetry or estimated by equations), thermic effect of food (assumed as 10% of TEE), and running cost (estimated by equations). The first week of the race, there were ~ 600 kcal/day from the measured TEE budget that could not be attributed to RMR, thermic effect of food, or running cost. This energy thus represented “other” unidentified physiological activities. By the last week of the race, measured TEE was almost fully explained by RMR, the thermic effect of food, and running cost. Thus, the energy expended in “other” physiological activities had decreased^21^. Based on our current data, we speculate that non-essential arm movements were part of the “other” activities measured the first week of the race. And as the distance ran accumulated, a compensation to reduce this non-essential activity (and probably others too) may have ensued. Notably, for the reallocation of energy from non-essential, non-muscular physiological activities to take place, a period of several weeks has been proposed^2^. In contrast, the compensation in the arm movements could be an earlier mechanism to constrain TEE.

In children, however, our data do not support a similar compensation as in adults. The reason for the difference in children vs. adults is unknown. A recent report showed similar TEE among children from industrialized (US/UK) and from forager-horticulturalist (Shuar) populations, thus supporting the constrained TEE model^7^. Therein, TEE budget was parsed into its classic components: RMR (measured by indirect calorimetry), thermic effect of food (assumed as 10% of TEE), and PA (the remainder, commonly named as *physical activity energy expenditure*). Intriguingly, even though hip-measured PA was higher in Shuar than US/UK children, the *physical activity energy expenditure* was higher in US/UK children^7^. Although factors such as movement efficiency may be involved, the result suggested that *physical activity energy expenditure* included energy from activities not attributed to hip-measured PA. We speculate that arm movements are among those activities. The fact that we have not observed a compensation in our current study may result from the children not being active enough. Regardless, strictly considering our current data, the high levels of locomotion (i.e. hip-measured PA) among Shuar children would not lead to a reduction of arm movements. Instead, locomotion may have led to decreases in other non-muscular, non-essential activities to maintain TEE within its physiological range. An attenuated circadian variation in RMR among Shuar children was proposed as an alternative^7^.

We conducted complementary analyses to gain insight into the association between locomotion and arm movements. As an attempt to isolate the arm movements, we adjusted wrist-measured PA for hip-measured PA. We then considered as total PA the sum of this wrist-measured PA_ADJ_ and hip-measured PA. As expected, total PA was directly associated with its components. But notably, the slope of the association was higher for wrist-measured PA_ADJ_ than for hip-measured PA, in both adults and children. These findings suggest that the individuals with the highest total PA were those with the largest dissociation between locomotion and arm movements. In other words, those individuals manifested the lowest compensation in arm movements. This capacity for dissociation may benefit health. Previous studies have shown that the energy expended in spontaneous PA (e.g. fidgeting) associates inversely with fat and weight gain^22,23^. Additionally, fidgeting has been associated with a better post-prandial glycemic response^24^, and also with a constitutional leanness phenotype^25^. Thus, individuals who can increase their locomotion without having a compensatory decrease in arm movements may manifest resistance to obesity and its associated complications. Future studies should test such hypothesis.

The constrained TEE model posits that, to sustain high PA levels, energy trade-offs should occur among the components of the TEE budget^2^. Evidence in adults suggests that the energy allocated to RMR does not participate in these trade-offs. Pontzer et al.^6^ found no evidence for an association between hip-measured PA and RMR (adjusted for confounding variables). Herein we confirmed those findings. We further showed no evidence for an association between RMR (adjusted for lean mass) vs. wrist-measured PA, wrist-measured PA_ADJ_, or total PA. Together, data suggest that physiological activities not accounted for by RMR are involved in the energy trade-offs. What proportion of this energy is explained by arm movements and by non-muscular activities is unknown. Future studies are required to fill this gap in knowledge.

The data analyzed herein are from studies designed for different purposes than our current analyses. Indeed, low levels of self-reported PA were among the inclusion criteria in both adult studies^9,10^. This limits the possibility of identifying a compensation in arm movements, which supposedly occurs among highly active individuals. Despite this limitation, we observed such a compensation above the 80th percentile of hip-measured PA. Notably, our sensitivity analyses in adults suggest that the compensation may occur only in males, but future studies should confirm this finding. In the case of the children cohort, PA levels were not among the inclusion/exclusion criteria^11^. Nevertheless, having excess body weight was an inclusion criterion, which may indicate that the children were not highly active. Also, previous data show that children from industrialized populations have lower hip-measured PA than children from forager-horticulturalist populations^7^. It is thus possible that we did not observe a compensation in arm movements because our children were not active enough. Finally, we speculate that high levels of locomotion result in decreases mostly of fidgeting, instead of other activities such as occupational activities. If there is a compensatory reduction in arm movements—as our adult data suggest–, those arm movements should be "non-essential". Certain occupational activities could be considered as "essential" for adults, e.g. typing on the computer for work; whereas moving the arms because of nervousness (an example of fidgeting) is not "essential". Nevertheless, note that fidgeting cannot be distinguished from occupational or household activities in our data.

In conclusion, we have shown that a compensatory reduction in arm movements may be involved in the constrained TEE model in adults. We speculate that at high levels of locomotion, there is a reduction in non-essential arm movements that, along with reallocation from yet unidentified physiological activities, allow locomotion to be sustained. The inclusion of both hip-worn and wrist-worn accelerometers will be valuable to better understand the constrained TEE model in future studies. This information will help to improve the effectiveness of interventions aimed at preventing and treating obesity.

## Methods

### Study design and subjects

The study was a cross-sectional analysis of the baseline measurements of previous clinical trials in adults and children. For the adult sample, we included two clinical trials involving similar experimental procedures (NCT02365129^9^ and NCT03334357)^10^. Adults were 18–65 years old, performed < 20 min of regular PA on 3 days/week (self-reported), had a stable body weight (< 3 kg change over the last 3 months), were not involved in weight loss programs, were non-smokers, took no medication, and suffered no chronic disease. No female subject was pregnant. Written informed consent was obtained from all subjects. The study protocols were approved by the Committee for Research Involving Human Subjects at the University of Granada (Spain; no. 924), the Servicio Andaluz de Salud (Centro de Granada, CEI-Granada, Spain), and the Human Research Ethics Committee of the Junta de Andalucía (Spain; no. 0838-N-2017). All the methods were performed in accordance with the declaration of Helsinki. The complete adult sample included 232 subjects, but only 211 met the inclusion criteria for accelerometer data (see "Physical activity" subsection). Moreover, two adults were excluded because they classified as extreme outliers for hip-measured PA (i.e. values higher than: Q3 + 3 × [Q3 − Q1]).

For the child sample, we included a single clinical trial (NCT02295072)^11^. Children were 8–12 years old, had overweight or obesity according to the World Obesity Federation criteria^26,27^, and had no physical disabilities or neurological disorders that limited exercise. No girl had started menstruating. Written informed consent was obtained from the parents of the children. The study protocols were approved by the Human Research Ethics Committee of the University of Granada (Spain; no. 848). All the methods were performed in accordance with the declaration of Helsinki. The complete children sample included 115 subjects, but only 105 met the inclusion criteria for accelerometer data (see "Physical activity" subsection).

### Body composition

Body weight and height were measured with an electronic scale and a stadiometer (SECA, Hamburg, Germany), with subjects wearing light underclothes. Whole-body fat mass and lean mass were measured by dual-energy X-ray absorptiometry following the manufacturer’s instructions (Discovery Wi DXA, Hologic, Inc., Bedford, MA, USA).

### Physical activity

Accelerometers (ActiGraph GT3X + , Pensacola, FL, USA) were worn on the non-dominant wrist and the right hip (on the iliac crest) for seven consecutive days. Subjects were required to remove the accelerometers only during water-based activities, and to register these non-wear time periods in a log. Sleeping times were also registered in the log. Accelerometers were initialized to record accelerations at 100 Hz with a dynamic range of ± 6 *G*. Raw accelerations were downloaded with the ActiLife software (ActiGraph, Pensacola, FL, USA) and then processed in the R package GGIR v.1.5.12^28^.

The processing steps in the GGIR were: (i) auto-calibration of the data according to local gravity acceleration^13^; (ii) detection of the non-wear time based on the raw acceleration recorded in the three axes^12^; (iii) detection of abnormally high accelerations related to malfunctioning of devices; (iv) calculation of the Euclidean norm minus one *G*, with negative values rounded to zero, aggregated in 5-s epochs^12^; (v) imputation of non-wear time and abnormal accelerations identified by using data from the rest of the days in the same time interval; (vi) identification of waking and sleeping time using an automated algorithm guided by the self-reported sleep logs^29,30^; and (vii) calculation of the average acceleration of all valid 5-s epochs on each day.

A day was considered as valid if it included ≥ 10 h of register during waking time, and ≥ 4 h of register during sleeping time. For the current analyses, we included only subjects with ≥ 4 valid days in both the wrist-worn and hip-worn accelerometers. Data from weekdays and weekends were weighed as: [(mean of weekdays × 5) + (mean of weekend days × 2) / 7]. Hip-measured PA and wrist-measured PA were expressed as milli-gravitational units (i.e. m*g*).

### Resting metabolic rate (RMR)

RMR was measured only in adults, and following recent recommendations^31^. Subjects were instructed to avoid moderate/vigorous activities during the 24–48 h before the test day. The evening before the measurement, the subjects consumed a standardized meal ad libitum. After a ~ 12-h overnight fast, subjects came to the lab in a motorized vehicle. RMR measurements were performed between 7:00 AM and 11:00 AM in a quiet room at 22–24 °C and 35–45% humidity. Subjects laid on a reclined bed in supine position for 20 min before beginning the measurement. During measurement, subjects were covered with a bed sheet and instructed to stay awake, breathe normally, avoid fidgeting and remain silent. Gas exchange (O_2_ consumption, CO_2_ production) was measured by indirect calorimetry over 30 min using a CPX Ultima CardiO2 or a CCM Express metabolic cart (Medical Graphics Corp, St. Paul, MN, USA). Data were processed using the MGCDiagnostic Breeze Suite 8.1.0.54 SP7 software (Medical Graphics Corp., St. Paul, MN, USA). Only the final 25 min of measurement were considered for the analysis, using the steady-state method for gas exchange data selection^32,33^. RMR (in kcal/day) was estimated using the Weir’s abbreviated Eq. ^34^.

### Statistical analyses

Data are presented as mean [standard deviation] (minimum − maximum). Data for adults and children were analyzed separately. IBM SPSS Statistics version 26 or GraphPad Prism version 8.4.2 were used for analyses. A *P* < 0.05 was considered significant.

The extra sum-of-squares F test was used to determine the regression model that fitted the data better; the null hypothesis was that a linear regression fitted the data better, while the alternative hypothesis was that a quadratic model did so. Pearson *r* values (2-tailed) and linear regression ß-coefficients [95% CI] were computed for subjects spanning variable hip-measured PA deciles: 1st to 10th, 2nd to 10th, 3rd to 10th, and so on.

To adjust wrist-measured PA for hip-measured PA, we calculated the residuals of the regression model that fitted the best the association between wrist-measured PA and hip-measured PA (i.e. quadratic model for adults, and linear model for children; Residual = measured value − predicted value). To avoid using positive and negative residual values when presenting the data, we added those residuals to the mean wrist-measured activity of the group, as previously done^6^. This new index—wrist-measured PA_ADJ_—is thus a surrogate of the accelerations produced by arm movements. Total PA was then calculated by adding hip-measured PA (i.e. locomotion) to wrist-measured PA_ADJ_ (i.e. arm movements). The slopes of the regressions hip-measured PA or wrist-measured PA_ADJ_ vs. total PA were compared with an F test.

A stepwise multiple linear regression was used to identify significant predictors of RMR in adults (n = 169). Seven candidate predictors were included: fat mass, lean mass, height, weight, body mass index, age and sex. After correcting for the family-wise error rate (*P*-value = 0.05/7 = 0.007), only lean mass appeared as a significant predictor (*P* < 0.001, R^2^ = 0.48). We thus computed an adjusted RMR—i.e. RMR_ADJ_—by adding the residuals from the “RMR ~ lean mass” linear regression to the mean RMR of the adults. The association of RMR_ADJ_ with the indexes of PA was assessed using the Pearson *r* test.

For continuous variables, differences between sexes or trials were tested with Student’s *t* test (2-tailed). Differences in the proportion of sexes between adult trials were tested with Chi-Square test.

## Supplementary Information

Supplementary Information.
