# Supplementary material for: Deciphering the constrained total energy expenditure model in humans by associating accelerometer-measured physical activity from wrist and hip
Source: Sci Rep. 2021 Jun 10;11:12302. doi: 10.1038/s41598-021-91750-x (PMC8192775; doi:10.1038/s41598-021-91750-x)
Supplement: Supplementary file 1 — Supplementary Information. [file 41598_2021_91750_MOESM1_ESM.pdf]

Supplementary information

Title: Deciphering the constrained total energy expenditure model in humans by associating accelerometer-measured physical activity from wrist and hip

Authors: Rodrigo Fernández-Verdejo, Juan M.A. Alcantara, Jose E. Galgani, Francisco M. Acosta, Jairo H. Migueles, Francisco J. Amaro-Gahete, Idoia Labayen, Francisco B. Ortega, Jonatan R. Ruiz

Figure S1

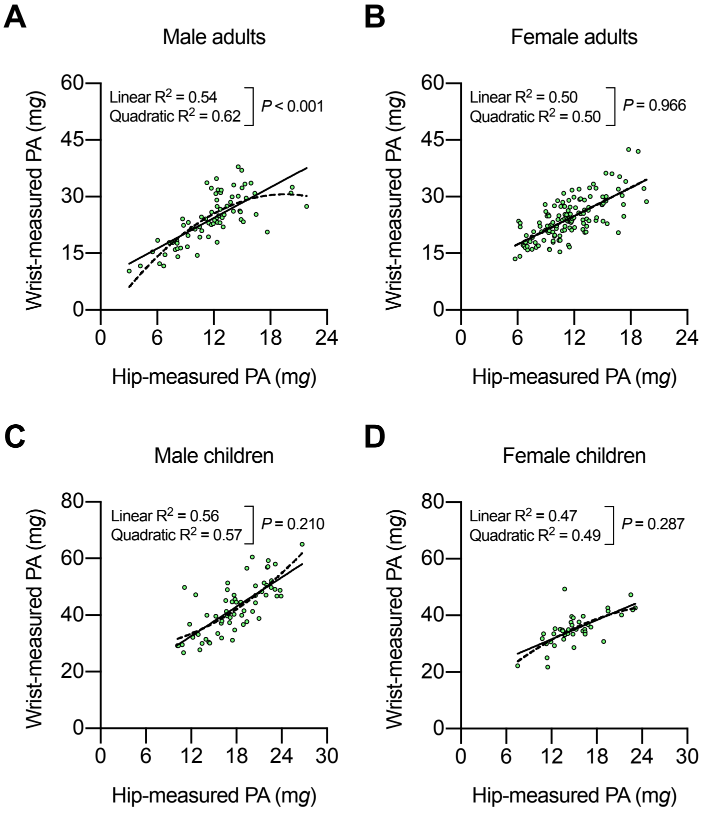

Figure S1. Association between wrist-measured and hip-measured physical activity (PA) stratified by sex. Quadratic [dashed line] and linear [solid line] regression models for the association in (A) male adults [n = 75], (B) female adults [n = 134], (C) male children [n = 63], and (D) female children [n = 42].

Figure S2

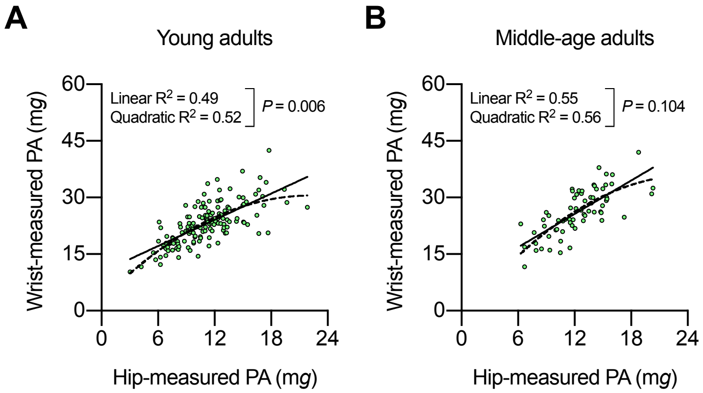

Figure S2. Association between wrist-measured and hip-measured physical activity (PA) stratified by trial (young adults, middle-age adults). Quadratic [dashed line] and linear [solid line] regression models for the association in (A) young adults [n = 143], and (B) middle-age adults [n = 66].

Table S1. Characteristics of the subjects stratified by sex.

	Adults		Children	
	Males	Females	Males	Females
n	75	134	63	42
Age, years	35.2 [16.2] (18.5 – 62.7]	30.3 [14.1] (18.2 – 66.0)*	10.1 [1.1] (7.9 – 11.9)	9.8 [1.0] (8.0 – 11.9)
Weight, kg	86.0 [14.9] (52.0 – 123.7]	64.8 [11.2] (45.0 – 99.9)***	57.1 [11.2] (36.7 – 84.7)	54.9 [10.8] (29.9 – 78.9)
Height, m	1.76 [0.06] (1.60 – 1.95]	1.63 [0.06] (1.48 – 1.80)***	1.44 [0.07] (1.27 – 1.66)	1.43 [0.08] (1.23 – 1.60)
Body mass index, kg/m^2	27.7 [4.6] (18.2 – 39.4]	24.3 [3.7] (17.2 – 34.3)***	27.0 [3.8] (20.8 – 37.8)	26.6 [3.4] (19.7 – 33.6)
Fat mass, kg[A]	28.3 [10.4] (10.0 – 51.7)	25.9 [7.8] (13.0 – 48.0)[#]	24.2 [6.5] (13.0 – 43.6)	25.2 [6.9] (12.1 – 41.1)
Lean mass, kg[A]	53.8 [6.9] (38.3 – 73.7)	35.7 [5.2] (22.7 – 50.4)***	30.2 [5.0] (21.7 – 46.1)	27.7 [5.2] (16.3 – 42.6)*
Resting metabolic rate, kcal/day[B]	1750 [327] (1108 – 2672)	1298 [205] (723 – 1795)***	Not measured	Not measured
Hip accelerometer				
Physical activity, mg	11.7 [3.5] (3.0 – 21.8)	11.4 [3.0] (5.7 – 19.6)	17.7 [3.9] (10.2 – 26.7)	15.0 [3.3] (7.5 – 23.1)**
Valid data, days	6.7 [0.5] (4.0 – 7.0)	6.8 [0.5] (5.0 – 8.0)	6.9 [0.5] (4.0 – 8.0)	6.8 [0.3] (5.0 – 7.0)
Non-wear time, h/day	0.37 [0.44] (0.00 – 1.98)	0.32 [0.40] (0.00 – 1.85)	0.18 [0.21] (0.00 – 0.85)	0.20 [0.18] (0.00 – 0.75)
Wrist accelerometer				
Physical activity, mg	24.0 [6.5] (10.2 – 37.9)	24.1 [5.3] (13.4 – 42.4)	42.5 [9.1] (26.7 – 65.0)	35.0 [5.5] (21.7 – 49.3)***
Valid data, days	6.7 [0.5] (4.0 – 7.0)	6.8 [0.5] (4.0 –8.0)	6.9 [0.4] (5.0 – 8.0)	6.9 [0.3] (6.0 – 8.0)
Non-wear time, h/day	0.33 [0.45] (0.00 – 2.47)	0.29 [0.39] (0.00 – 2.00)	0.17 [0.23] (0.00 – 1.07)	0.15 [0.16] (0.00 – 0.69)

Data are mean [standard deviation] (minimum – maximum). [A]n = 62 in male children; [B]n = 63 in male adults, and 106 in female adults. [#]$P < 0.10$, *$P < 0.05$, **$P < 0.01$, ***$P < 0.001$ vs. males in the same age group.

Table S2. Characteristics of the adults stratified by trial.

	Adults	
	Young	Middle-age
n	143	66
Males/Females, n	45/98	30/36[$]
Age, years	22.1 [2.2] (18.2 – 26.6)	53.7 [5.1] (44.9 – 66.0)***
Weight, kg	71.0 [16.8] (45.0 – 123.7)	75.6 [14.5] (50.6 – 107.2)[#]
Height, m	1.67 [0.08] (1.52 – 1.95)	1.67 [0.09] (1.48 – 1.89)
Body mass index, kg/m^2	25.0 [4.6] (17.2 – 39.4)	26.7 [3.5] (18.8 – 34.8)**
Fat mass, kg	25.2 [8.9] (10.0 – 51.7)	30.1 [7.8] (15.5 – 51.7)***
Lean mass, kg	41.7 [9.8] (28.1 – 73.7)	43.2 [11.8] (22.7 – 63.6)
Resting metabolic rate, kcal/day[A]	1442 [308] (846 – 2672)	1505 [378] (723 – 2633)
Hip accelerometer		
Physical activity, mg	11.1 [3.2] (3.0 – 21.8)	12.4 [3.0] (6.2 – 20.3)**
Valid data, days	6.7 [0.5] (5.0 – 7.0)	6.7 [0.5] (4.0 – 8.0)
Non-wear time, h/day	0.38 [0.44] (0.00 – 1.98)	0.24 [0.34] (0.00 – 1.46)*
Wrist accelerometer		
Physical activity, mg	23.1 [5.3] (10.2 – 42.4)	26.2 [6.0] (11.6 – 41.9)***
Valid data, days	6.7 [0.5] (4.0 – 7.0)	6.8 [0.6] (4.0 – 8.0)
Non-wear time, h/day	0.34 [0.43] (0.00 – 2.47)	0.21 [0.35] (0.00 – 2.00)*

Data are mean [standard deviation] (minimum – maximum). [A]n = 103 in young adults. [$]$P < 0.10$ vs. young adults; [#]$P < 0.10$, *$P < 0.05$, **$P < 0.01$, ***$P < 0.001$ vs. young adults.
